# Frequency of *G6PD Mediterranean* in individuals with and without malaria in Southern Pakistan

**DOI:** 10.1186/s12936-017-2069-4

**Published:** 2017-10-24

**Authors:** Bushra Moiz, Haroon Muhammad Arshad, Ahmed Raheem, Hasan Hayat, Najia Karim Ghanchi, M. Asim Beg

**Affiliations:** 1 Department of Pathology and Laboratory Medicine, The Aga Khan University Hospital, Karachi, Pakistan; 2 Aga Khan Medical College, Karachi, Pakistan; 3 Haematology & Transfusion Medicine, Karachi, Pakistan

**Keywords:** G6PD deficiency, Falciparum malaria, Malaria, Blood donors

## Abstract

**Background:**

Pakistan has an estimated annual burden of 1.5 million malaria cases. The current situation calls for an effective malaria control and eradication programme in this country. Currently, primaquine is an attractive option for eliminating reservoirs of *Plasmodium vivax* hypnozoites and killing gametocytes of *Plasmodium falciparum*. However, this drug causes haemolysis in individuals who are glucose-6-phosphate (G6PD) deficient. It is important to map G6PD deficiency and malaria distribution in Pakistan to design an effective malaria eradication regimen. Frequency of G6PD deficiency (G6PDd) in malaria patients has not been reported from Pakistan in any meaningful way. The purpose of this study was to evaluate the frequency of *G6PD c.563C*>*T* (*G6PD Mediterranean*) in male individuals with and without falciparum malaria.

**Methods:**

Two hundred and ten archived DNA samples from males (110 from falciparum malaria patients and 100 from healthy individuals) were utilized in this study. Healthy blood donors were selected based on stringent pre-defined criteria. Patients were confirmed for malaria parasites on microscopy and or immune chromatographic assay detecting *P. falciparum* histidine-rich protein 2. Parasitaemia was also computed. DNA samples were tested for *G6PD c.563C*>*T* mutation through PCR–RFLP according to the previously defined protocol and its allelic frequency was computed.

**Results:**

*G6PD c.563C*>*T* was observed in four of 110 patients with falciparum malaria and in two of 100 healthy donors. Mean (± SD) haemoglobin, median (IQR) platelet and median (IQR) parasite count in G6PD-deficient malaria-patients were 8.9 ± 0.9 g/dL, 124 × 109/L (IQR 32, 171) and 57,920/μL of blood (IQR 12,920, 540,000) respectively.

**Conclusions:**

Cumulative allelic frequency for *G6PD 563c.C*>*T* was 0.0285 detected in 6 of 210 X-chromosomes in Southern Pakistan. Frequency for this *G6PD* allele was 0.0364 in malaria-patients and 0.0200 in healthy individuals. Large studies including females are needed to elucidate the true burden of G6PDd in malaria-endemic areas. The information will enable local health policy makers to design effective strategies for eliminating malaria form this region.

## Background

With an estimated load of 1.5 million malaria cases each year, Pakistan is among seven countries of the WHO Eastern Mediterranean Region sharing 95% of the total regional malaria burden [1]. Malaria with *Plasmodium vivax* is endemic and more common (88%) in this country, while malaria with *Plasmodium falciparum* is seen only during rainy seasons or post rain and accounts for 12% of the malaria burden [2]. The current situation calls for an effective malaria control and eradication programme in Pakistan. Currently, the only drug that is available for malaria eradication is primaquine (an 8-aminoquinoline) which kills *P. vivax* hypnozoites [3] and *P*. *falciparum* gametocytes [4]. However, the administration of primaquine may be dangerous in individuals who are glucose-6-phosphate dehydrogenase (G6PD) deficient.

G6PD is a pentose pathway enzyme which catalyzes the rate limiting step in the reduction of nicotine amide adenine dinucleotide phosphate (NADP). Reduced NADP is critical for eradicating free radicals from the cells and maintaining their viability. Therefore, generally asymptomatic, G6PD-deficient individuals are at risk of drug-, food- or infection-induced acute haemolytic anaemia. Because the *G6PD* gene is located on the X-chromosome, the prevalence of G6PD in males is higher than that of females. Interestingly, global distribution of G6PD deficiency (G6PDd) matches that of past and present malaria [5], suggesting a genetic advantage regarding malaria risk. For example, in Africa, *G6PD A*-type offers relative protection against falciparum malaria in hemizygous males and to some extent in heterozygous females [6]. Similarly, *G6PD Mediterranean* (*G6PD*-*Med*)—a variant common in Asian countries confers immunity against vivax malaria [7] in hemizygous males.

Previous work has shown that G6PD deficiency (G6PDd) is widely prevalent in Northern Pakistan with a frequency ranging from 2 to 8% in various ethnic groups [8]. Moreover, *G6PD*-*Med*, is the most common G6PD variant in Pakistan [9]. Ashley et al. reported life threatening haemolysis on primaquine administration in individuals carrying *G6PD*-*Med* [10]. This indicates that primaquine administration for malaria eradication requires mandatory G6PD testing in Pakistan. Since this test is not widely available, malaria will continue to be an economic burden for the country.

There is limited information for G6PD-malaria interaction in Pakistan raising several research questions: What is the prevalence of G6PDd in Southern Pakistan? Does *G6PD*-*Med* protect hemizygous males and heterozygous or homozygous females from *P. falciparum*? To respond to these basic questions, large-scale studies are needed. The present study was aimed in determining allelic frequency of *G6PD c.563C*>*T* in male individuals with or without malaria by using archived DNA samples. This small-scale study is anticipated to become a basis for larger studies in evaluating the true burden of G6PDd before planning mass primaquine therapy.

## Methods

### Setting

This was a descriptive study conducted in 2013 at a tertiary care academic institute, Aga Khan University Hospital (AKUH), Karachi, Pakistan. Karachi is the largest city of Pakistan and is the residence to several ethno-lingual groups from all over the country. Muhajirs (emigrants from India) constitute 50% of its population, while Pashtuns are the second largest group. Other ethnic groups include Sindhis, Punjabis, Bengalis, Anglo-Indians, Parsi and Catholics. AKUH because of its location caters mostly Karachites. Therefore, it can be anticipated that ethnic background of individuals visiting AKUH or its blood bank will be homogenous. This is more rightly so, as blood bank works on the principle of exchange donations thereby prompting relatives of admitted patients to donate blood. Approximately 40,000 subjects donate blood annually at AKUH blood bank of which 99% are male donors. The total number of admissions with malaria in AKUH between January 2009 and December 2011 was 365 [11] with estimated annual admissions of 122 patients with malaria. Clinical laboratories at Aga Khan University received over 15,000 microscopy requests for malarial parasites and subsequently report approximately 1800 positive cases each year.

### Subjects

#### Selection of participants

Two hundred and ten archived DNA samples were utilized in the study. This included 110 males who were infected with falciparum malaria during 2006–2007 and 100 healthy males who donated blood at AKUH during the same time period. Random sample identification technique was used to select patients from a large pool of archived DNAs. The focus was to determine G6PDd allelic frequency in males who were infected with falciparum malaria (less common malaria in Pakistan) for which we had limited archived samples in AKUH DNA bank. EDTA blood sample was collected at the time patient presented with malaria, DNA extracted and archived at − 80 °C in the research laboratory for future usage. Healthy blood donors were selected based on stringent pre-defined criteria for blood donation such as no history of malaria in past 3 years. G6PD deficiency is an x-linked disorder and mainly affects males, therefore, only males were included in this study. Moreover, national data for G6PDd was available for males predominantly. In addition, there is a preponderance of male blood donors in Pakistan—the control group in this study. Female patients and all other patients suffering from vivax malaria were excluded.

#### Malaria diagnosis

For blood donors, ICT malaria test was used for screening malaria. Patients were routinely diagnosed on microscopy (of thick and thin films) and or immune chromatographic assay (ICT) for *P. falciparum* histidine rich protein2 (HRP2). Briefly, thick blood films were air dried and dipped in water to remove haemolyzed red cells. Slides were then stained with 4–5% Giemsa with phosphate buffer saline (pH 7.2) for 25 min and then rinsed with tap water. Thick films were examined microscopically under high power field (100× oil immersion lens) for parasitized erythrocytes against 200 white blood cells. Parasite density was estimated on thick film assuming a standard value of 8000 white blood cells per microlitre of blood. Parasitaemia was calculated as follows:

Number of parasites = Total parasite counted × 200 white cells/No of white cells/µL of blood. Thin peripheral smears were stained with Leishman’s stain and reviewed at 40× and 100× objectives.

#### Clinical details

Medical charts were reviewed for various clinical (respiratory and nervous system manifestations) and laboratory features (complete blood count, parasitaemia, liver function tests, serum creatinine and electrolytes) were retrieved from the computerized laboratory data system. Duration of hospital stay was also determined. Patients were divided into paediatric and adult groups and the cut off was ≤ 17 and > 17 years, respectively, as per institutional policy.

#### Mutational analysis

Archived DNA samples from malaria patients (n = 110) and blood donors (n = 100) were tested for *G6PD c.563C*>*T* mutation through previously defined protocol [9]. Other reported variants like *G6PD Chatham* were not studied as the study was focused on determining relation between *G6PD*-*Med* (predominant and severe form) and *P. falciparum* (having high morbidity and mortality).

#### Ethical concerns

Ethical approval for this study was granted by The Aga Khan University Ethical Review Committee (#509-Pat/ERC-06). Informed consent was taken from blood donors and malaria patients for genotyping. Re-coding was done to assure anonymity of the subjects.

#### Data analysis

Data was entered in Microsoft Excel sheet and transferred to SPSS version 22 (IBM Statistic data, USA) for statistical analysis. Frequency was calculated for age and *G6PD* variant. Descriptive analysis (mean ± SD with range) was computed for quantitative data with normal distribution and median and inter-quartile range (IQR) for skewed data. Laboratory parameters were compared with respect to age group and the threshold of significance was below 0.05. *G6PD* allele frequency was computed as number of affected males divided by total number of males.

## Results

### Demographic findings

One hundred and ten malaria-infected males with a mean age of 27.2 ± 17.5 years were studied. Twenty-nine % of patients were children below 17 years of age. The blood donors were young males of mean age 27 ± 7.0 years (range 17–52).

### Clinical details

Clinical details were available for only 48 malaria patients (7 children and 41 adults) who were hospitalized. Significant clinical findings included fever (n = 110, 100%), splenomegaly (n = 13, 27%) and pulmonary oedema (n = 3). Six adults demonstrated complications like cerebral malaria (n = 3), respiratory failure (n = 2) and renal failure (n = 1). Mean stay (in days) in hospital and intensive care was 4.38 ± 4.33 and 0.89 ± 2.99 days respectively. No mortality was observed in any patient. Treatment included artemisinin (n = 4), doxycycline (n = 8), quinine (n = 4), and combination of anti-malarial in rest of the patients. Blood donors recruited in the study had no history of malaria in past 3 years. Two patients carrying G6PD mutation did not show complicated malaria and received artemisinin plus doxycycline and quinine.

### Laboratory findings

Laboratory details were available for all malaria patients (n = 110). Eighty-two percent patients were anaemic, 14.5% were severely anaemic (with haemoglobin < 7 g/dL), 66% patients had thrombocytopaenia while 12% were leucopaenic. Twenty patients had haemoglobinuria as reported in urine analysis. Laboratory parameters with respect to age group are summarized in Table 1). Relatively low platelets, more parasitaemia and higher creatinine were observed in adults compared to children. Blood donors recruited in the study were negative for ICT malaria test and had haemoglobin above 12.5 g/dL as per the blood bank policy. G6PD assay was not performed in any individual.

**Table 1:** Demographic, clinical and laboratory data for paediatric and adult patients with malaria (n = 110)

Parameters	Paediatric group	Adult group	All patients
n	32	78	110
Age in years (mean ± SD)	6.9 ± 4.9	35.2 ± 13.9	27.0 ± 17.6
Haemoglobin g/dL (mean ± SD)	9.2 ± 2.7	10.7 ± 2.8	10.3 ± 2.8
White cell count × 10^9^/L(median; IQR)	8.0 (5.9–9.3)	6.9 (4.8-10.8)	7.3 (5.0–10.0)
Platelet count × 10^9^/L(median; IQR)	130 (105–255)	109 (55–151)	120 (59–183)
Parasite count/µL (median; IQR)	2916 (800–26,180)	15,740 (4185–59,290)	9960 (2360–48,740)
Alanine aminotransferase IU/L (median; IQR)	47 (42–71)	43.9 (31.0–53.0)	44.7 (32.53)
Serum creatinine (mg/dL) (median; IQR)	0.9 (0.6–1.2)	1.3 (1.0–1.6)	1.2 (0.9–1.4)

### Molecular studies

*G6PD 563c.C*>*T* was detected in four of 110 patients with falciparum malaria and in two of 100 healthy blood donors. Cumulative allelic frequency for *G6PD 563c.C*>*T* was 0.0273 detected in 6 of 220 X-chromosomes (Table 2). There were two pediatric (age 5 and 12 years) and two adult patients (age 33 and 36 years) who were identified carrying *G6PD 563c.C*>*T.* Mean (± SD) haemoglobin, median (IQR) platelet and median (IQR) parasite count in G6PD-deficient malaria-patients were 8.9 ± 0.9 g/dL, 124 × 109/L (IQR 32, 171) and 57,920/μL of blood (IQR 12,920, 540,000), respectively.

**Table 2:** *G6PD c.563C*>*T* allele frequency in the study group (n = 210)

Subjects	n	G6PD563C>T	Allelic frequency
Healthy donors	100	02	0.0200
Malaria patients	110	04	0.0364
All individuals	210	06	0.0285

## Discussion

This study showed an allelic frequency of 3.6% for *G6PD*-*Med* in subjects infected with malaria in Southern Pakistan and 2.8% in all tested individuals. This is lower than average allelic frequency reports for G6PD deficiency from various regions of Pakistan. But considering that only one G6PD mutation (*G6PD 563c.C*>*T*) was looked for, we would have captured 80% of the G6PDd prevalence. Other G6PD variants reported from Pakistan were G6PD Orissa and Chatham [9], for which gene-primaquine interaction is not known [12]. Literature review showed that there were 15 national surveys [13–27] with an average of 200 individuals per survey. Studies done so far from 1966 to date showed a frequency of 3.9% (range 1.1–8.5%) for G6PDd in Northern Pakistan. Highest frequency was observed in Pashtun males as 5.3% compared to Punjabis (3.3%), Sindhis (2.7%) and Mohajir (2.2%) males (Table 3). This G6PDd frequency is comparable with global G6PD map computed by Howes et al. in 2012 [28]. The data for females was not analyzed as they were underrepresented in all studies.

**Table 3:** Allele frequency for *G6PDd* in multi-ethnic males in Pakistan [13–25, 27]

Ethnic groups	Male N (%)	Deficient male N (% of total)	Allelic frequency
Pashtun	1321 (17.0)	84 (6.4)	0.0636
Punjabi	3227 (43.5)	71 (2.2)	0.0220
Sindhi	397 (5.4)	11 (2.8)	0.0277
Baloch	NA	NA	NA
Bengali	146 (2.0)	2 (1.4)	0.0137
Kashmiri	460 (6.2)	5 (1.1)	0.0109
Mohajir	45 (0.6)	1 (2.2)	0.0222
Miscellaneous	1817 (24.5)	115 (6.3)	0.0633
Total	7750 (100)	592 (3.9)	0.0390

*NA* not available

Gething et al. in 2011 estimated *P. falciparum* transmission globally through mathematical modeling [29]. Accordingly, 1.13 and 1.44 billion people, respectively, were at risk of stable and unstable malaria worldwide in 2010. In this model, Punjab, Gligit Baltistan, some areas of Baluchistan and Sindh were depicted as having a low annual incidence of < 1 malaria case in a population of 10,000 [30]. Khyber Pakhtunkhwa (KPK) and majority of Sindh were evaluated to have stable malaria transmission having a prevalence of > 1:10,000 population [30]. There is also high transmission of malaria in districts bordering Afghanistan and Iran that carry 37% of national malaria burden with an annual incidence exceeding 4.5 cases/1000 population [31]. Interestingly, largest number studies to date are from KPK which show a high incidence of G6PD deficiency with an allele frequency of 0.241. This indirectly indicates that G6PD deficiency does not provide absolute immunity against infection with *P. falciparum*. This was observed in the current study as well, as four of 110 patients with falciparum malaria had *G6PD 563c.C*>*T* mutation and were not immune to malaria. Moreover, the G6PDd malaria patients had high parasite burden of 152,821.00 ± 259,297.01/μL in this study. In contrast, Guindo et al. reported protective effect of *G6PD A*-*type* in African males against falciparum infection [6]. The variance may be due to predominance of *G6PD 563c.C*>T in Pakistani population that may have different effect on *P. falciparum* survival than that observed in *G6PD A*-African population. More studies are needed on the immunity (if any) conferred by *G6PD 563c.C*>T against *P. falciparum* in hemizygous males and heterozygous females.

*G6PD 563c.C*>*T* is highly prevalent in West Asia including Pakistan [12]. This mutation causes severe haemolysis when challenged with primaquine. This may be life threatening leading to renal failure and death [12]. The effect of primaquine therapy on females who are carriers for this mutation is not known. Currently, safe alternatives for primaquine therapy are not available [3]. Therefore, mandatory G6PD testing is the only option for Pakistan before intervening for malaria eradication with primaquine. More studies are needed on genome sequencing for identifying G6PD variants in Pakistan. This information can be used in developing an in-house point-of-care testing device for phenotype-genotype analysis and correct mapping of G6PDd. It can be incorporated in the mandatory neonatal screening as is available for hypothyroidism in Pakistan.

## Strength and limitations

The study provides a database for *G6PD 563c.C*>*T* variance in Southern Pakistan demonstrating presence of this variant in patients suffering from malaria. The study had certain limitations as mutational analysis in females and in patients suffering from vivax malaria was not done. G6PD assay was not done hence the true prevalence of G6PD deficiency was underestimated. Moreover, other G6PD mutations except *G6PD 563c.C*>*T* were not identified. However the study did provide a snapshot for the presence of severe form of G6PD deficiency in Southern Pakistan.

## Conclusions and recommendations

Cumulative allelic frequency for *G6PD 563c.C*>*T* was 0.0273 detected in 6 of 220 X-chromosomes. The allele frequency is in line with previously reported data from Pakistan. This study advocates mandatory need for G6PD testing prior to anti-malarial intervention with primaquine therapy. Escalating doses of primaquine in patients with G6PD deficiency needs to be addressed in future studies.

## Abbreviations

AKU-ERC: Aga Khan University ethical review commitee
EDTA: ethylenediaminetetraacetic acid
ERC: ethical review committee
DNA: de-oxyribonucleic acid
G6PD: glucose-6-phosphate dehydrogenase
G6PDd: glucose-6-phosphate dehydrogenase deficiency
ICT: immunochromatography
PCR: polymerase chain reaction
RFLP: restriction fragment length polymorphism
SPSS: statistical package of social sciences
WHO: World Health Organization

## References

[1] WHO/EMRO. Reported malaria cases in countries with high malaria burden. Cairo: World Health Organization; http://www.emro.who.int/annual-report/2012/table-2-reported-malaria-cases-in-countries-with-high-malaria-burden.html. Assessed 30 May 2017.

[2] Khattak AA, Venkatesan M, Nadeem MF, Satti HS, Yaqoob A, Strauss K (2013). Prevalence and distribution of human Plasmodium infection in Pakistan. Malar J.

[3] Baird JK, Hoffman SL (2004). Primaquine therapy for malaria. Clin Infect Dis.

[4] WHO. Single dose primaquine as a gametocytocide in *Plasmodium falciparum* malaria. Updated WHO policy recommendation (October 2012). 2012. p. 1. http://www.who.int/malaria/pq_updated_policy_recommendation_en_102012.pdf. Assessed 30 May 2017.

[5] Nkhoma ET, Poole C, Vannappagari V, Hall SA, Beutler E (2009). The global prevalence of glucose-6-phosphate dehydrogenase deficiency: a systematic review and meta-analysis. Blood Cells Mol Dis.

[6] Guindo A, Fairhurst RM, Doumbo OK, Wellems TE, Diallo DA (2007). X-linked G6PD deficiency protects hemizygous males but not heterozygous females against severe malaria. PLoS Med.

[7] Leslie T, Briceno M, Mayan I, Mohammed N, Klinkenberg E, Sibley CH (2010). The impact of phenotypic and genotypic G6PD deficiency on risk of *Plasmodium vivax* infection: a case–control study amongst Afghan refugees in Pakistan. PLoS Med.

[8] Moiz B (2013). A review of G6PD deficiency in Pakistani perspective. J Pak Med Assoc.

[9] Moiz B, Nasir A, Moatter T, Naqvi ZA, Khurshid M (2011). Molecular characterization of glucose-6-phosphate dehydrogenase deficiency in Pakistani population. Int J Lab Hematol.

[10] Ashley EA, Recht J, White NJ (2014). Primaquine: the risks and the benefits. Malar J.

[11] Zubairi ABS, Sobia N, Afsheen R, Vikram M, Anita Fazal R, Najia Karim G (2013). Severe *Plasmodium vivax* malaria in Pakistan. Emerg Infect Dis.

[12] Howes RE, Dewi M, Piel FB, Monteiro WM, Battle KE, Messina JP (2013). Spatial distribution of G6PD deficiency variants across malaria-endemic regions. Malar J.

[13] Saleem A (1966). Glucose-6-phosphate dehyrogenase deficiency and hemolytic anemia. J Pak Med Assoc.

[14] Ronald AR, Underwood BA, Woodward TE (1968). Glucose-6-phosphate dehydrogenase deficiency in Pakistani males. Trans R Soc Trop Med Hyg.

[15] Stern MA, Kynoch PA, Lehmann H (1968). Beta-thalassaemia, glucose-6-phosphate-dehydrogenase deficiency, and haemoglobin D-Punjab in Pathans. Lancet.

[16] McCurdy PR, Mahmood L (1970). Red cell glucose-6-phosphate dehydrogenase deficiency in Pakistan. J Lab Clin Med.

[17] Hashmi JA, Farzana F, Ahmed M (1976). Abnormal hemoglobins, thalassemia trait & G6PD deficiency in young Pakistani males. J Pak Med Assoc.

[18] Khattak M, Dawood M, Saleem M (1992). The prevalence of glucose-6-phosphate dehydrogenase deficiency in Northern Pakistan. Pak Armed Forces Med J.

[19] Saha N, Ramzan M, Tay JS, Low PS, Basair JB, Khan FM (1994). Molecular characterisation of red cell glucose-6-phosphate dehydrogenase deficiency in north-west Pakistan. Hum Hered.

[20] Bouma MJ, Goris M, Akhtar T, Khan N, Khan N, Kita E (1995). Prevalence and clinical presentation of glucose-6-phosphate dehydrogenase deficiency in Pakistani Pathan and Afghan refugee communities in Pakistan; implications for the use of primaquine in regional malaria control programmes. Trans R Soc Trop Med Hyg.

[21] Khan M (2004). Glucose 6 phosphate dehydrogenase deficiency in adults. J Coll Physicians Surg Pak.

[22] Ali N, Anwar M, Ayyub M, Bhatti FA, Nadeem M, Nadeem A (2005). Frequency of glucose-6-phosphate dehydrogenase deficiency in some ethnic groups of Pakistan. J Coll Physicians Surg Pak.

[23] Rasheed S, Hayee A, Lodhi Y, Ahmed R (2005). Neonatal jaundice and glucose 6 phosphate dehydrogenase deficiency. Ann King Edw Med Coll.

[24] Khan TA, Ahmed S, Anwar M, Ayyub M (2008). The frequency of glucose-6-phosphate dehydrogenase deficiency in Punjabis and Pathans. JPMI.

[25] Mehmood A, Akhtar M, Niazi MFK (2010). Frequency of glucose-6-phosphate dehydrogenase deficiency in asymptomatic Pakistani population. Pak Armed Forces Med J.

[26] Khan ZR, Shaheen N, Ali N (2014). The elusive cases of carrier females and G6PD deficiency. Int J Pathol.

[27] Khan ZR, Najeeb S, Amjad A (2015). G6PD deficiency: glucose 6 phosphate dehydrogenase deficiency; the case for mass screening in Pakistan. Prof Med J.

[28] Howes RE, Piel FB, Patil AP, Nyangiri OA, Gething PW, Dewi M (2012). G6PD deficiency prevalence and estimates of affected populations in malaria endemic countries: a geostatistical model-based map. PLoS Med.

[29] Gething PW, Patil AP, Smith DL, Guerra CA, Elyazar IR, Johnston GL (2011). A new world malaria map: *Plasmodium falciparum* endemicity in 2010. Malar J.

[30] MAP. The spatial limits of *Plasmodium falciparum* malaria transmission map in 2010 in Pakistan. 2011. http://www.map.ox.ac.uk/browse-resources/transmission-limits/Pf_limits/PAK/. Accessed 30 Apr 2017.

[31] Kakar Q, Khan MA, Bile KM (2010). Malaria control in Pakistan: new tools at hand but challenging epidemiological realities. East Mediterr Health J.

